# Supplementary material for: EnzML: multi-label prediction of enzyme classes using InterPro signatures
Source: BMC Bioinformatics. 2012 Apr 25;13:61. doi: 10.1186/1471-2105-13-61 (PMC3483700; doi:10.1186/1471-2105-13-61)
Supplement: Addtional file 5 — The Java code to format the data files, evaluate and predict. The file enzml_java_code.tar.gz contains the Java code used to format database data to ARFF and XML formats, to execute cross and train-test (jackknife) evaluations and to record evaluation results to database. More information is included in the readme.txt file and the Javadoc files. The code can be used with a MySQL database. To use a different database software, other JDBC drivers might be required. [file 1471-2105-13-61-S5.gz › java_code/utils/doc/uk/ac/ed/inf/utils/stats/PseudoTruncatedParetoSingleton.html]

PseudoTruncatedParetoSingleton


---


|  |  |  |
| --- | --- | --- |
| **Overview** \| **Package** \| **Class** \| **Use** \| **Tree** \| **Deprecated** \| **Index** \| **Help** | |  |
| **PREV CLASS**   **NEXT CLASS** | **FRAMES**    **NO FRAMES**     **All Classes** |  |
| SUMMARY: NESTED \| FIELD \| CONSTR \| METHOD | DETAIL: FIELD \| CONSTR \| METHOD |  |


---


## uk.ac.ed.inf.utils.stats Class PseudoTruncatedParetoSingleton

```
java.lang.Object
  uk.ac.ed.inf.utils.stats.PseudoTruncatedParetoSingleton
```

---

``` public class PseudoTruncatedParetoSingleton extends java.lang.Object ```

Class

**Version:**
:   4 Aug 2008

**Author:**
:   Luna De Ferrari luna.deferrari-at-ed.ac.uk

---

| **Method Summary** | |
| --- | --- |
| `static PseudoTruncatedPareto` | `getInstance(double scale, double shape, int offset, int truncationThreshold, int lengthOfPseudoRandomList)` |
| `PseudoTruncatedPareto` | `getPseudoTruncatedPareto()` |

**Methods inherited from class java.lang.Object**

`equals, getClass, hashCode, notify, notifyAll, toString, wait, wait, wait`

**Method Detail**

### getPseudoTruncatedPareto

```
public PseudoTruncatedPareto getPseudoTruncatedPareto()
```

---


### getInstance

```
public static PseudoTruncatedPareto getInstance(double scale,
                                                double shape,
                                                int offset,
                                                int truncationThreshold,
                                                int lengthOfPseudoRandomList)
```


---


|  |  |  |
| --- | --- | --- |
| **Overview** \| **Package** \| **Class** \| **Use** \| **Tree** \| **Deprecated** \| **Index** \| **Help** | |  |
| **PREV CLASS**   **NEXT CLASS** | **FRAMES**    **NO FRAMES**     **All Classes** |  |
| SUMMARY: NESTED \| FIELD \| CONSTR \| METHOD | DETAIL: FIELD \| CONSTR \| METHOD |  |


---
